# What Do We Know about Barley miRNAs?

**DOI:** 10.3390/ijms232314755

**Published:** 2022-11-25

**Authors:** Adriana Volná, Martin Bartas, Petr Pečinka, Vladimír Špunda, Jiří Červeň

**Affiliations:** 1 Department of Physics, University of Ostrava, 710 00 Ostrava, Czech Republic; 2 Department of Biology and Ecology, University of Ostrava, 710 00 Ostrava, Czech Republic; 3 Global Change Research Institute, Czech Academy of Sciences, 603 00 Brno, Czech Republic

**Keywords:** gene expression, regulation, barley, miRNAs, plants, environmental stress

## Abstract

Plant miRNAs are powerful regulators of gene expression at the post-transcriptional level, which was repeatedly proved in several model plant species. miRNAs are considered to be key regulators of many developmental, homeostatic, and immune processes in plants. However, our understanding of plant miRNAs is still limited, despite the fact that an increasing number of studies have appeared. This systematic review aims to summarize our current knowledge about miRNAs in spring barley (*Hordeum vulgare*), which is an important agronomical crop worldwide and serves as a common monocot model for studying abiotic stress responses as well. This can help us to understand the connection between plant miRNAs and (not only) abiotic stresses in general. In the end, some future perspectives and open questions are summarized.

## 1. Introduction

MicroRNAs (miRNAs) are important players in post-transcriptional gene expression regulation in multicellular species. miRNAs can modify/decrease the expression of fully or partially complementary mRNA molecules [1,2]. Original reports about RNA silencing, where miRNAs belong, date back to the 1990s [3] when the first attempt to introduce a chimeric chalcone synthase using *Agrobacterium tumefaciens* vector led to the decrease or complete loss of anthocyanin pigmentation in petals (flowers) of *Petunia hybrida* [4]. A similar observation was in 1992 documented also for the *Neurospora crassa* where the transformation using plasmids containing artificial constructs led to the albino phenotype [5]. Another experiment in 1993 carried out by Victor Ambros, Rosalind Lee and Rhonda Feinbaum resulted in the final revelation that the *lin-4* gene (involved in the regulation of *Caenorhabditis* elegans developmental events) codes not for a protein, but for small RNA with regulatory function [6], and such RNAs were later called miRNAs and their nomenclature was established [7]. Today, miRNAs are considered to be master regulators of many cell differentiation, developmental, and homeostatic processes in animals [8] and plants [9,10], and are also widely accepted as an important component of the cellular immune system, which is documented even for plant species [11,12,13,14].

Despite the fact that the number of studies dealing with plant miRNAs during the past few years has steeply increased (from 11 articles in 2002 to the current 928 in 2021), our complex understanding of their role in environmental stress responses remains limited, see, e.g., current review dealing with miRNA regulation and stress adaptation in plants [15]. Our specific focus on barley stems from the importance of this agronomical crop, which was one of the first cultivated grains as early as 10,000 years ago [16]. Barley is currently ranked 4th in worldwide production after wheat, maize, and rice, and is extensively used in food production, for feeding cattle, or brewery worldwide [17,18]. Additionally, barley plants serve as an important commodity with potential health benefits—from hail to barley grass food supplements. Effects on the gut microbiota and suppression of already developed chronic diseases including obesity, diabetes, circulatory disorders, and cancer were documented or at least hypothesized [19,20]. An additional and equally important application of barley seedlings is its use as a monocot model species (often considered as a model plant for the whole *Triticeae* tribe). Barley has a short cultivation period from seed planting to sampling the material (14 days) and does not require complex growth conditions which makes it a suitable species for a wide range of experiments [21,22,23]. Lastly, barley has a sequenced reference genome (cultivar Morex, NCBI ID: GCF_904849725.1) of a length of 4.27 Gbps consisting of 7 chromosomes, and circular chloroplastic DNA [24]. More than one-half of all 58,438 predicted genes (53%; total of 31,449) are protein-coding genes, while approximately one-tenth are pseudogenes (9.9%; total of 5778) [25]. Most importantly, almost one-third of the barley genes are coding for small RNAs (30.3%; total of 17,729) [25] which further highlights their functional relevance in gene expression regulation and justifies the need to properly understand their involvement in physiological and stress-related processes. The rest of the genes are uncharacterized (6%; a total of 3481) [25]. For the above-mentioned reasons, we decided to perform a review focused specifically on spring barley (*Hordeum vulgare*) miRNAs. In the following chapters, we will briefly discuss plant miRNAs biogenesis and regulatory potential in general, then we will move to specific roles of miRNAs in barley physiology and stress responses, conserved barley miRNAs and their high-confidence mRNA targets, and finally give some possible future directions of research in this field, with focus on barley.

## 2. Plant miRNAs—Biogenesis and Regulatory Potential

**Figure 1:**
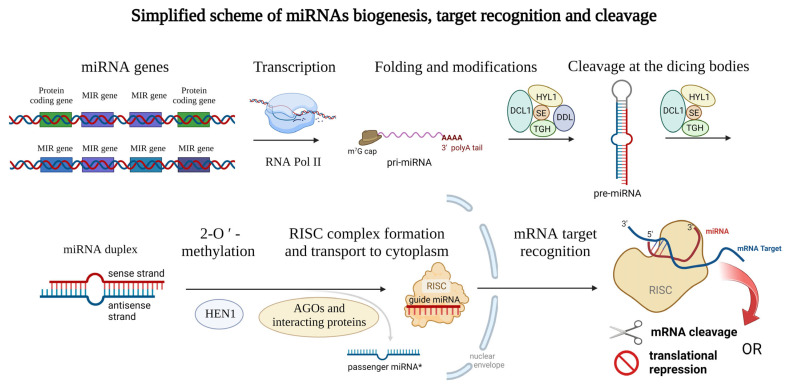
Schematic representation of the miRNAs biogenesis. Genes encoding miRNAs are transcribed by RNA polymerase II and modified on their ends (m^7^G cap and polyA tail) and thus the primary microRNA (pri-miRNA) arise. Then, the typical stem-loop structure is formed by complementary base pairing and cleaved at the dicing bodies (consisting of several proteins including DCL1, HYL1, SE, TIGH, and DDL) resulting in miRNA duplex formation which can be later 2′-O-methylated (ensured by the HEN1 protein). Guide miRNA is incorporated into the RISC consisting of several proteins, and transported into the cytoplasm, where mRNA target recognition and cleavage can take place while the passenger miRNA is released away. Proteins from the Argonaute family (AGOs) can modify the stability of the miRNAs and also affect the interaction with target mRNAs. This figure was created using BioRender (https://biorender.com/; accessed on 20 June 2022).

Mature plant miRNAs are 19–25-nucleotide-long ribonucleic acids that can have either intergenic (miRNA gene is localized between two protein-coding sequences of the DNA) or intragenic origin [26], where miRNAs are cleaved from the mRNA sequences during the splicing (also called intron-derived miRNAs [27]). Specifically, in barley, more than 75% of miRNAs are transcribed from intergenic loci [28]. The biogenesis of miRNAs is ensured by the DNA-dependent RNA polymerase II which is responsible for the biosynthesis itself [2]. In some cases, multiple plant miRNAs are synthesized all at once (multiple miRNAs localized in one long transcript) [29] and often form a miRNA family, which is a group of miRNAs derived from a common ancestor [30]. Emerging miRNAs can be modified co-transcriptionally, or post-transcriptionally. Similar to other transcripts, a 7-methylguanosine (m^7^G) cap is attached to the 5′ end of the miRNA, and the 3′ end is polyadenylated (or can be spliced) [31]. Later, the transcript encoding miRNA (or multiple miRNAs) is folded to the stem-loop structure which is called pri-miRNA [2] (meaning primary miRNA transcript). Such pri-miRNAs are further cleaved by the dicing bodies. Dicing bodies consists of several proteins including DICER-LIKE 1 (DCL1), DAWDLE (DDL), HYL1, TGH, and SE [32,33], resulting in miRNA duplex formation which can be later 2′-O-methylated by the HEN1 methylase [34] and incorporated into the RNA-induced silencing complex (RISC) [2,35]. The complex issue of further proteins involved in plant miRNA biogenesis is reviewed in Li et al., 2021 [36]. miRNAs of both origins (intragenic as well as intergenic) lead to the formation of a mature RISC with incorporated mature miRNA. In most cases, only the sense/guide miRNA strand is incorporated into the RISC, while the antisense/passenger miRNA (miRNA*) strand is disrupted, but recently also the regulation potential of the passenger miRNA became the center of interest [14,37,38]. For a clear summary of miRNA biogenesis see Figure 1 below.

miRNAs interact with their target mRNAs mostly at their 3′ UTRs, but interactions occurring in the 5′ UTRs or coding regions were documented as well [39,40]. RISC is directed to the complementary mRNA transcript, whereby the Watson–Crick base-pairing aligns guide miRNA and target mRNA transcript, and depending on the central miRNA region complementarity, mRNA is cleaved (usually when there is perfect base-pair complementarity), or translation repression occurs (central miRNA region is not completely complementary to mRNA) [41]. Moreover, in the case the target mRNA is cleaved, so-called phased secondary small interfering RNAs (phasiRNAs) can arise [42]. phasiRNAs are 21 or 24-nucleotide-long siRNAs having important roles in plant stress responses [42], development [43], and reproduction [44].

Similar to the other genes, miRNA transcription is precisely fine-tuned. This is assured mainly by transcription factors binding [45] and methylation status of DNA [46], both heavily influenced by endogenous and exogenous stimuli. In 2018, protein WHIRLY1 was found to be involved in increased levels of nuclear miRNAs in high-light conditions in barley. It was therefore proposed that WHIRLY1 can bind to RNA and it might be a general factor influencing the biogenesis and/or stability of various miRNAs [47].

An additional level of miRNA complexity is their dynamic stability [33,48]. It was documented that the processes such as 3′-end modifications and interaction with Argonaute proteins (AGOs) can both reduce and increase the stability of miRNAs depending on the actual needs of the plant. For example, AGO1 from *Arabidopsis thaliana* was proposed to stabilize miRNAs, and miRNA–mRNA target interaction [2].

Besides post-transcriptional gene silencing (PTGS), miRNAs can regulate plant genes via RNA-induced methylation of DNA [49,50]. Such a process was in detail described in the *Arabidopsis thaliana*, where miRNAs (miR165, miR166) regulate the methylation status of *PHB* and *PHV* genes [51], and are responsible for the determination of the abaxial and adaxial leaf side. Similarly, the miRNA-induced gene methylation was described even in the *Oryza sativa* where the miR1873 ensures the methylation of its own gene [50]. To make our understanding of miRNAs-based regulation of gene expression more challenging, the stimulative effect of miRNAs on gene expression was observed and documented as well [52].

Last but not least, in 2015 it was proposed that plant pri-miRNAs are capable of encoding small functional peptides [53,54] described as miPEPs. The best-characterized miPEPs (miPEP171d, miPEP172c, and miPEP858a) were found in plant species including *Arabidopsis thaliana* (miPEP165a [53], miPEP858 [55]), *Medicago truncatula* (miPEP171b [41]), *Glycine max* (miPEP172c [56]), and *Vitis vinifera* (miPEP171d1 [57]). The mechanism of miPEPs molecular function is still largely unclear, but generally, miPEPs positively affect the accumulation of their associated miRNAs [54]. It is also likely that many of miPEPs will be species-specific [57].

## 3. miRNAs in Barley Physiology and Stress Responses

**Figure 2:**
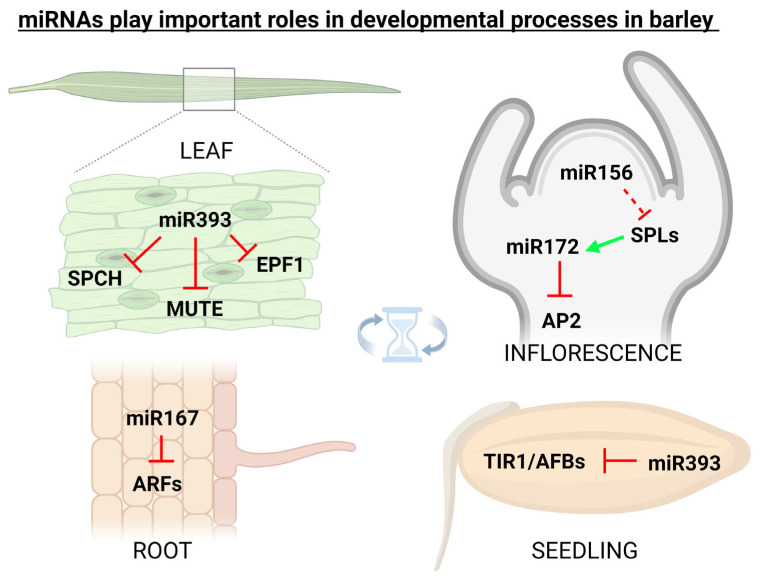
miRNAs play important roles also in the developmental processes. In spring barley (*Hordeum vulgare*), specific miRNAs were linked with the targets involved in the regulation of flowering, root development, seed germination, and also with stomata development. Inhibition is indicated by the red ┴ mark, while positive effect by the green arrow. This figure was created using BioRender (https://biorender.com/; accessed on 20 June 2022).

miRNAs in plants are important regulators of various physiological processes including shoot apical meristem development [58], leaf growth [59], flower formation [60], seed production [61], and root expansion [59]. It was found that miRNA171 in barley is responsible for the regulation of shoot meristem development through three independent pathways, i.e., firstly through the down-regulation of SCARECROW-LIKE (SCL) transcription factors, secondly via up-regulation of miRNA156 and repressing vegetative phase transitions (a possibly monocotyledon-specific mechanism), and thirdly by repressing expression of *TRD* and *HvPLA1* genes [62]. Additionally, flower development in grasses including barley is tightly regulated by miRNAs. It was found that miRNA159, miRNA171, miRNA172, and miRNA396 regulate the expression of floral organ identity genes in barley, rice, and maize [63]. In barley, cleistogamous flowering (i.e., shedding its pollen before opening) arises from the suppression of the AP2 transcription factor via miR172, originally thought to be a result of target mRNA cleavage [64], but later it was proved that miR172-mediated AP2 regulation occurs at the translational level [65]. It is also known that the expression of barley miR393 is active in the developmental period, and its misexpression affects seedling growth and stomatal density [66]. In 2018, it was found that miR160 in barley simultaneously targets class II ARF members which are functionally involved in developmental stages by regulating the auxin-mediated genes [67]. Figure 2 illustratively depicts some of the most known barley miRNAs (and their targets) that play important roles in developmental processes.

Besides the non-stress conditions, miRNAs play key roles in gene expression regulation in response to a variety of abiotic stimuli, including several stress responses. In plants, their involvement in many abiotic stress responses including heat stress responses, low-temperature responses, drought exposure responses, carbon dioxide responses, light stress responses, or gamma radiation responses was reported [68,69,70,71,72]. Specifically in barley, miRNAs responsive to salinity stress [73,74,75,76], drought [77,78,79,80,81], nitrogen [82], boron [83], phosphorus [84,85], aluminum [74,86,87], cadmium [88], cold deacclimation [89], heat stress [90], and possibly to light [21] were identified till date. A chronological summary of the most impactful miRNA studies in barley (starting from 2010) can be found below in Table 1.

**Figure 3:**
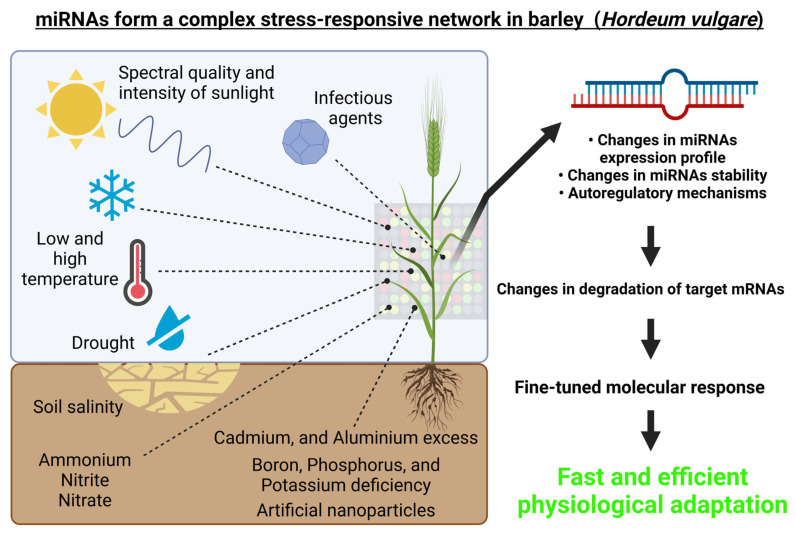
miRNAs form a complex regulatory network in barley (*Hordeum vulgare*). Environmental cues, both abiotic (i.e., spectral quality and intensity of the incident light, growth temperature, drought, high salinity, heavy metals exposure, etc.) and biotic (for example pathogens) can affect the expression of miRNAs and thus also their target genes. This figure was created using BioRender (https://biorender.com/; accessed on 20 June 2022).

From the above-mentioned studies, it is evident that barley miRNAs play a complex role in responses to various abiotic and biotic stresses or stimuli, which is schematically depicted in Figure 3.

## 4. Target Transcripts of Barley miRNAs

Several web-based tools, resources, and databases related to small RNAs comprising miRNAs in plants exist [106]. To browse miRNAs identified in barley to date, mainly PNRD [107], PmiREN [108], miRBase [109], Plant small RNA genes [110], or the integrative miRNEST database [111] can be used. As these databases use slightly different methods of required miRNAs evidence or data sources, overall counts of miRNAs deposited here differ. Total counts of barley miRNAs in each of the databases together with direct links and other useful information are listed in Table 2.

According to TarDB: A miRNA Target Database in Plants [112] (http://www.biosequencing.cn/TarDB/browse.html, accessed 20 June 2022), there are currently 20 conserved miRNAs in barley (Table 3). It is worth mentioning that TarDB uses relatively strict parameters to identify high-confidence plant miRNAs and their targets based on cross-species conservation filter, degradome, and sRNA-seq data, so Table 3 below is rather illustrative than exhaustive. It is supposed that the overall number of functional miRNAs in barley is much higher, according to a study published in November 2021 [113], a total of 156 miRNAs including 35 known and 121 novel miRNAs experimentally identified in Tibetan hull-less barleys, targeting over 1200 genes (nonetheless it was done by computational prediction, only selected targets were also in vitro verified using RLM-5′ RACE method) [113].

**Figure 4:**
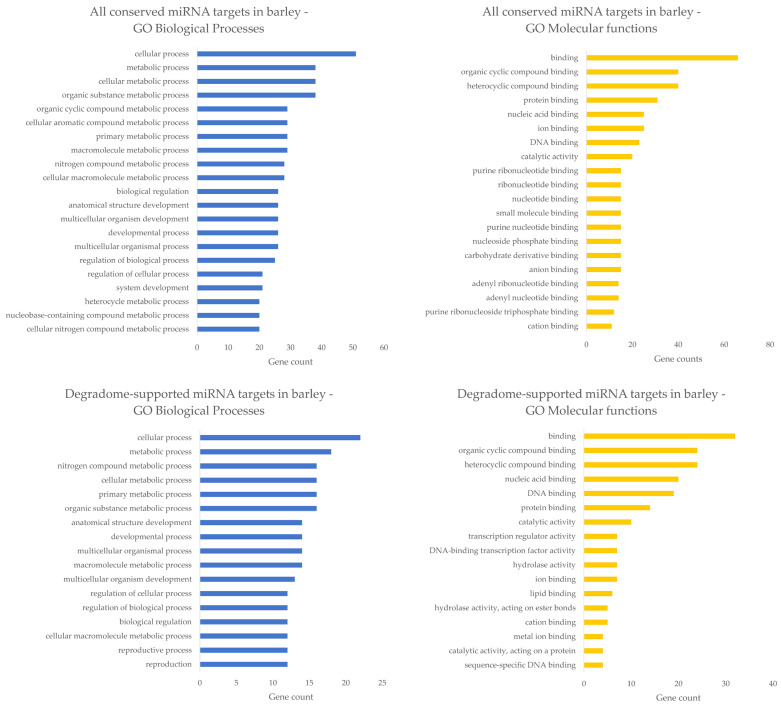
Biological processes and Molecular functions of miRNA targets in barley. In the upper half of the image, the most abundant GOs of all conserved miRNA targets in barley are shown. In the lower half of the image, the most abundant GOs of degradome-supported miRNA targets are depicted. Blue bar plots stand for biological processes, whereas the orange ones correspond to molecular functions.

Many barley miRNAs are targeting mRNA transcripts encoding transcription factors. This is maybe not too surprising, as it was previously known that most of the plant miRNA targets are transcription factors that regulate plant growth and development [135]. In Figure 4, known barley miRNA targets from TarDB [112] are grouped according to their gene ontologies (GOs), separately for ‘all conserved miRNA targets’ (dataset containing 92 mRNAs) and ‘degradome-supported miRNA targets’ (dataset containing 37 mRNAs, 15 of them are common with the first subset of ‘all conserved miRNA targets’). GO terms for these datasets were acquired using PLAZA Workbench [136,137]. It can be seen that most miRNA targets participate in diverse biological processes, comprising metabolic, developmental, regulatory, and reproductive processes. This fact may be in good aggreement with general observations and knowledge from miRNA studies not only in barley plants. From the point of molecular functions view, barley miRNA targets are employed mainly in binding processes (e.g., organic cyclic compound binding, protein binding, nucleic acid binding, etc.) and some targets possess catalytic activity (Figure 4). It is worth noting that the above-mentioned GOs are more general than specific, and they deserve more detailed analysis in the future. Moreover, it may be interesting that many miRNA targets (once translated into proteins) can bind DNA and theoretically act as transcriptional activators or repressors influencing the expression of their superior miRNA genes, thus forming another regulatory layer (or feedback loop) [138]. This issue could certainly serve as a potential theme for further research in the field of plant development and stress responses.

It is essential to bear in mind that a particular miRNA can interact with many different mRNA molecules [139], and that particular miRNA targets can be relatively quickly acquired through plant evolution [140]. A good example is miR168a from sweet orange (*Citrus sinensis* L. Osbeck), where besides its original target (AGO1 mRNA) it gained a novel target, CUC2 mRNA [141]. Another specific case was observed in rice (*Oryza sativa japonica* cv. Nipponbare), where miR159 triggers MAP kinase 8 mRNA, in addition to its original target (MYB mRNA) [142]. Interestingly, miRNA activity can be regulated by bait in the form of long non-coding RNAs (lncRNAs), where such lncRNAs mimic the target mRNAs and sequester specific miRNAs (preventing them from interacting with their mRNA targets)—this phenomenon is usually described as (mi)RNA decoy [143] or Target Mimics [144]. In barley, there is a study from 2020 where authors identified about 8000 lncRNAs and found a total of 32 endogenous target mimics that may potentially decoy 18 different miRNAs [145].

**Figure 5:**
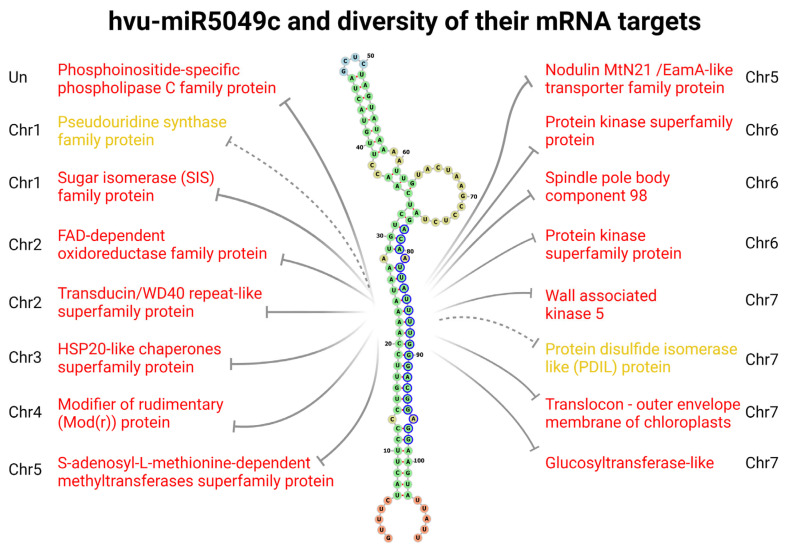
Barley pri-miR5049c structure together with miR5049 putative mRNA targets. pri-miRNA structure was computed via RNAfold web server [149] and visualized in the form of a Forna diagram [150]. Nucleotides in blue circles correspond to the mature 21nt-long miRNA region. Grey lines depict inhibition of specific mRNA targets (if the line is full and the description is in red, mRNA cleavage was predicted, according to TarDB: “Cleavage is predicted if miRNA 5′ positions 9–11 have the perfect match”). Chromosome numbers correspond to the location of genes encoding particular mRNAs, Un stands for Unplaced locus.

As an illustrative example of miRNAs targets diversity, we depicted all computationally predicted mRNA targets of single barley miRNA, particularly miR5049c (Figure 5). According to TarDB [112], this miRNA has the potential to target 17 different mRNAs originating from various genes across the whole barley genome (Chromosomes 1 to 7). The molecular and biological functions of proteins encoded by these mRNAs are also very diverse, and some of them participate in response to external stimuli, e.g., HSP20-like chaperones superfamily protein (by homology) [146]. The fact that one miRNA can bind multiple mRNA targets is relatively well-known for many years [147,148]. Obviously, at the same time, a single miRNA molecule can bind only a single mRNA target, therefore one may imagine that the relative accessibility of particular mRNA to a particular miRNA determines the proportion of specific mRNA-miRNA interactions. The cell usually produces only a fraction of all possible mRNAs, and therefore such a mechanism of regulation would seem efficient.

## 5. Conclusions and Future Directions

This review gives a basic overview of a rapidly growing amount of miRNA studies in barley (*Hordeum vulgare*). From what we know, it is clear that miRNAs play an important role in many developmental processes as well as in a variety of stress-induced molecular and biological responses. It is likely that more and more putative miRNAs will be discovered in barley, and many of them will be linked to abiotic or biotic stresses, including drought, cold, high temperatures, high salinity, micronutrient excess or deficiency in the soil, spectral quality of incident light, or infectious agents. Identification of plant miRNA targets on a large scale has traditionally been made mainly by bioinformatic approaches [151,152,153,154,155,156]. On the other hand, experimental validation is needed to verify predicted mRNA targets—historically, this has been done using laboratory-intensive in vitro methods like the 5′ RACE assay [157], but nowadays, the high-throughput degradome sequencing technique can be employed to validate (at least partially) predicted miRNA targets. Nonetheless, four criteria (according to a nice review by Giulia Riolo et al. [158]) should ideally be fulfilled when validating novel miRNAs:

(a) Showing co-expression of miRNA and target mRNA in vivo;
(b) Proving interaction between miRNA and a specific site within target mRNA;
(c) Demonstrating miRNA-mediated effects on target protein expression;
(d) Demonstrating miRNA effects on biological function.

What is quite difficult for our complex understanding of miRNA mechanisms is that even different genotypes/cultivars of barley tend to express unique miRNA patterns. This may point to rapid miRNA evolution allowing gene expression fine-tuning in a dynamically changing environment and agriculture. In addition, different plant tissues may express a different ‘miRNome’ in response to various stress signals [159].

Several studies have discussed the possibility of miRNA-based technology to improve plant resistance to abiotic factors [160,161]. In 2017, Jannatul Ferdous et al. published a study where the drought-inducible expression of miR827 enhanced drought tolerance in transgenic barley [162]. In maize, the knock-down of miR166 using short tandem target mimics technology resulted in enhanced abiotic stress resistance, abscisic acid level elevation, and indole acetic acid level reduction [163]. As miR166 is conserved also in barley, it would be interesting to identify whether its knock-down would have similar effects. Another promising possibility offers CRISPR/Cas technology already utilized for miRNA gene editing in rice [164,165] and *Arabidopsis thaliana* [166], further reviewed in [167]. Finally, there is an increasing effort to use exogenous/artificially made miRNAs (or siRNAs) in modern plant protection and improvement, and such RNA interference technology is usually considered GMO-free [168]. Among the various options, chitosan nanoparticles bearing miRNAs seem to be particularly attractive [169].

Barley miRNAs could also be efficiently used as molecular markers. In 2020, researchers proposed selected miRNAs as a tool to monitor the barley response to soil compaction [170].

Below, several outstanding questions are summarized:

(1) Are some of the barley miRNAs tissue/developmental, or stage-specific? Are we able to catalog it in some integrative and user-friendly way? For this purpose, it would be beneficial to have something like a barley miRNA atlas (similar to PmiRExAt, where wheat, rice, maize, and Arabidopsis miRNAs in multiple tissues and developmental stages can be found) [171].
(2) Which barley miRNAs have the potential to become a useful stress biomarker? In other words, do some stress-specific miRNAs exist?
(3) Is barley miRNome rather complete, or not? Compared to rice, wheat, and Arabidopsis, the total number of known barley miRNAs is still lack behind, and *bona fide* many discoveries waiting for us!

**Figure 6:**
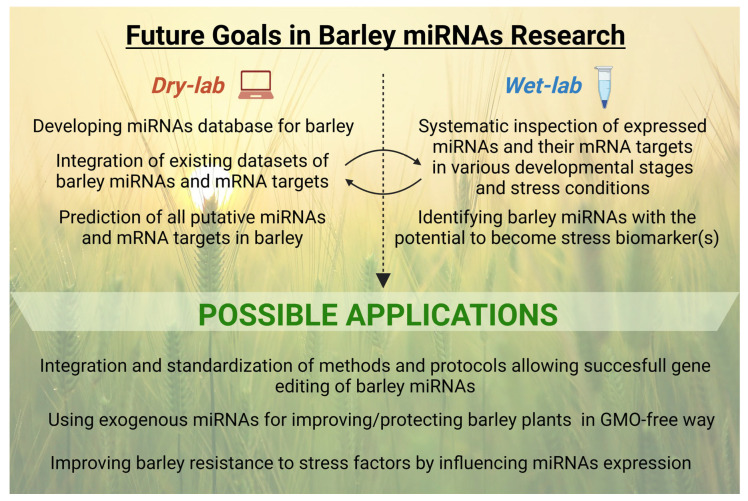
Future goals in miRNAs research in barley, divided into dry-lab and wet-lab categories, and possible future applications.

To better depict the above-mentioned perspectives in barley miRNAs research, we have created a diagram where particular future aims are divided into two categories, i.e., work to be done either using dry-lab or wet-lab methods, together with possible future applications (Figure 6).

All in all, even though a lot is known about miRNAs in barley, much remains to be resolved. Aristotle said “the more you know, the more you realize you don’t know”, and complex miRNAs problematics in barley (and generally in plants) could definitely fit this quote.

## Figures and Tables

**Table 1:** Chronological summary of studies dealing with miRNAs in barley species and most important results obtained.

Title of the Study and Reference	Barley Cultivars Inspected	Year of Publication	Most Important Findings
Regulation of barley miRNAs upon dehydration stress correlated with target gene expression [79]	*Hordeum vulgare*	2010	A total of 28 potential miRNAs were identified using bioinformatic approaches (BLASTn of known plant miRNAs and barley expressed sequence tags (ESTs), and RNA folding algorithms).
Discovery of barley miRNAs through deep sequencing of short reads [91]	*Hordeum vulgare* cultivars Golden Promise and Pallas	2011	The first large-scale study of miRNAs in *Hordeum Vulgare*, 100 miRNAs were identified (only 56 of them had orthologs in wheat, rice, or Brachypodium) and 3 candidates were validated in vitro using a Northern blot assay.
Identification and Characterization of MicroRNAs from Barley (*Hordeum vulgare* L.) by High-Throughput Sequencing [92]	*Hordeum vulgare* L.	2012	126 conserved miRNAs (belonging to 58 families), and 133 novel miRNAs (50 families) were identified in this study.
miRNA regulation in the early development of barley seed [61]	*Hordeum vulgare*	2012	84 known miRNAs and 7 new miRNAs together with 96 putative miRNA target genes were identified during the early development of barley seeds (first 15 days post anthesis).
Developmentally regulated expression and complex processing of barley pri-microRNAs [93]	*Hordeum vulgare* cultivar Rolap	2013	miRNA genes in barley often contain introns which may play important role in miRNA processing.
A Comprehensive Expression Profile of MicroRNAs and Other Classes of Non-Coding Small RNAs in Barley Under Phosphorous-Deficient and -Sufficient Conditions [84]	*Hordeum vulgare* L., cultivar Pallas	2013	221 conserved miRNAs and 12 novel miRNAs were identified, many of them were phosphorus condition-specific. A total of 47 miRNAs were significantly differentially expressed between the two phosphorus treatments.
Boron Stress Responsive MicroRNAs and Their Targets in Barley [83]	*Hordeum vulgare* L. cultivar Sahara	2013	31 known and 3 new miRNAs were identified in barley, and 25 of them were found to respond to boron treatment.
Transcriptionally and post-transcriptionally regulated microRNAs in heat stress response in barley [90]	*Hordeum vulgare* cultivar Rolap	2014	Four heat stress up-regulated barley miRNAs were found (miR160a, miR166a, miR167h, and miR5175a).
Differential expression of microRNAs and other small RNAs in barley between water and drought conditions [80]	*Hordeum vulgare* cultivar Golden Promise	2014	Three novel miRNAs, designated as hvu-miRX33, hvu-miRX34, and hvu-miRX35 were identified. hvu-miRX34 had no homologous miRNA in wheat.
The miR9863 Family Regulates Distinct Mla Alleles in Barley to Attenuate NLR Receptor-Triggered Disease Resistance and Cell-Death Signaling [94]	*Hordeum vulgare* L.	2014	The key role of the miR9863 family in the immune response to the pathogen (powdery mildew fungus, *Blumeria graminis* f. sp. *hordei*) was proposed
Polycistronic artificial miRNA-mediated resistance to Wheat dwarf virus in barley is highly efficient at low temperature [95]	Artificially transformed *Hordeum vulgare* cultivar Golden Promise	2015	Polycistronic artificial miRNA in plasmid vector was successfully transformed into barley embryos and mediated resistance to Wheat dwarf virus.
Global Identification of MicroRNAs and Their Targets in Barley under Salinity Stress [73]	*Hordeum vulgare* cultivar Morex	2015	Authors identified 152 miRNAs (142 conserved and 10 novel ones), and 44 miRNAs (39 conserved and 5 novel ones) were found to be salinity-responsive.
Characterization of microRNAs and their targets in wild barley (*Hordeum vulgare* subsp. spontaneum) using deep sequencing [96]	*Hordeum vulgare* subsp. *spontaneum*	2016	A total of 70 known miRNAs and 18 novel miRNA candidates were identified and many of them were predicted to target mRNAs encoding transcription factors.
Developmental changes in barley microRNA expression profiles coupled with miRNA target analysis [97]	*Hordeum vulgare* cultivar Rolap	2016	miRNA transcriptomes of five barley developmental stages were inspected. Overall, miR168-3p and miR1432-5p levels increased while the 5′U-miR156-5p level decreased during barley development.
miR393-Mediated Auxin Signaling Regulation is Involved in Root Elongation Inhibition in Response to Toxic Aluminum Stress in Barley [86]	*Hordeum vulgare* cultivar Golden Promise	2017	Barley miR393 was functionally characterized. It regulates root sensitivity to aluminum through the alteration of auxin signaling.
Differential expression of microRNAs and potential targets under drought stress in barley [78]	*Hordeum vulgare* L. cultivars Commander, Fleet, Hindmarsh, and breeding line WI4304	2017	miRNA regulation under drought stress in barley is genotype-specific.
microRNAs participate in gene expression regulation and phytohormone cross-talk in barley embryo during seed development and germination [98]	*Hordeum vulgare* cultivar Golden Promise	2017	A total of 1324 known miRNAs and 448 novel miRNA candidates were identified. miR393-mediated auxin response regulation significantly affected grain development.
Small RNA Activity in Archeological Barley Shows Novel Germination Inhibition in Response to Environment [99]	Ancient *Hordeum vulgare*	2017	Sequencing of miRNAs obtained from archeological barley samples (600–900 years BP) revealed their local adaptation to an agrarian environment around the river Nile.
Genome-wide analysis of the SPL/miR156 module and its interaction with the AP2/miR172 unit in barley [100]	*Hordeum vulgare* L.	2018	The study identified 17 barley *SPL* genes, and 7 of them contain a putative miR156 target site.
Identification of microRNAs in response to aluminum stress in the roots of Tibetan wild barley and cultivated barley [87]	*Hordeum vulgare* Al-sensitive Golden Promise and Tibetan wild barley (Al-tolerant XZ29)	2018	50 miRNAs responsive to aluminum stress were detected, and some of them were found to be exclusively expressed in Al-tolerant XZ29.
Identification of microRNAs responding to salt stress in barley by high-throughput sequencing and degradome analysis [76]	Tibetan wild barley accession XZ16; *Hordeum vulgare* cultivar Golden Promise	2019	miR393a, miR156d, and miR172b (regulating *HvAFB2/HvTIR1*, *UGTs*, and *HvAP2*) are responsible for salt tolerance in barley roots.
Genotypic difference of cadmium tolerance and the associated microRNAs in wild and cultivated barley [88]	*Hordeum vulgare* cultivar Golden Promise and wild barley WB-1	2019	216 conserved miRNAs (in 59 miRNA families) and 87 novel miRNAs were identified. Authors suggest that miRNAs may play critical roles underlying the genotypic difference of cadmium tolerance in barley.
Genome-Wide Identification and Characterization of Drought Stress Responsive microRNAs in Tibetan Wild Barley [81]	Tibetan wild barley *Hordeum vulgare* L. ssp. Spontaneum	2020	69 conserved miRNAs and 1574 novel miRNAs were identified, some of them were differentially expressed in drought conditions.
Barley microRNAs as metabolic sensors for soil nitrogen availability [82]	*Hordeum vulgare* cultivar Golden Promise	2020	Authors identified 13 barley miRNAs that are nitrogen excess responsive with the possible function of metabolic sensors for soil nitrogen availability.
The Impact of Zinc Oxide Nanoparticles on Cytotoxicity, Genotoxicity, and miRNA Expression in Barley (*Hordeum vulgare* L.) Seedlings [101]	*Hordeum vulgare* L. var. Abava	2020	ZnO nanoparticles significantly changed the expression of barley miR156a, miR159a, and miR159c in a dosage-dependent manner.
Identification of microRNAs in response to low potassium stress in the shoots of Tibetan wild barley and cultivated [102]	A Tibetan wild barley accession (XZ153) and a cultivar (ZD9) differing in low K tolerance	2021	A total of 1088 miRNAs were identified in the two barley genotypes under low potassium conditions. 65 of them were significantly differentially expressed.
Barley Seeds miRNome Stability during Long-Term Storage and Aging [103]	*Hordeum vulgare* cultivar Damazy	2021	miRNome of barley seeds harvested in 1972 was inspected. 61 known and 81 novel miRNA were identified pointing to the fact that miRNAs in dry seeds are extremely stable.
Identification microRNAs and target genes in Tibetan hulless barley to BLS infection [104]	*Hordeum vulgare* L. variety nudum Hook. f.	2021	A total of 36 conserved and 56 novel miRNAs were identified, some of them were differentially expressed between BLS (barley leaf stripe fungal disease)-sensitive and BLS-tolerant barley genotypes.
Pi-starvation induced transcriptional changes in barley revealed by a comprehensive RNA-Seq and degradome analyses [85]	*Hordeum vulgare* L.	2021	Authors suggest that barley adapts to inorganic phosphate (Pi)-starvation also via differential expression of several miRNAs.
Identification of microRNAs Responding to Aluminium, Cadmium and Salt Stresses in Barley Roots [74]	*Hordeum vulgare* cultivar Golden Promise	2021	525 miRNAs (198 known and 327 novel miRNAs) were identified through high-throughput sequencing. 31 miRNAs were differentially expressed under inspected stresses.
An miR156-regulated nucleobase-ascorbate transporter 2 confers cadmium tolerance via enhanced anti-oxidative capacity in barley [105]	*Hordeum vulgare* genotypes Zhenong8 (ZN8) (Cd-tolerant genotype) and W6nk2 (Cd-sensitive genotype)	2022	miR156g-3p_3 targets a novel nucleobase-ascorbate transporter gene (*HvNAT2*). *HvNAT2* evolved from the *Zygnematales* in *Streptophyte algae* and positively regulates cadmium tolerance → genetic engineering of NAT in plants may have potential in the remediation of soil/water cadmium pollution
Regulation of Phenolic Compound Production by Light Varying in Spectral Quality and Total Irradiance [21]	*Hordeum vulgare* L. cultivar Bojos	2022	Several barley miRNAs were differentially expressed in response to the spectral quality of incident light.

**Table 2:** User-friendly online databases comprising barley miRNAs.

Database Name	Direct Link	The Overall Count of Barley miRNAs	Notes
Plant Non-coding RNA Database (PNRD)	http://structuralbiology.cau.edu.cn/PNRD/index.php	71	58 of them were experimentally validated
Plant MicroRNA Encyclopedia (PmiREN)	https://www.pmiren.com/	178	Divided into 94 miRNA families
miRBase	https://www.mirbase.org/summary.shtml?org=hvu	69	/
Plant small RNA genes	https://plantsmallrnagenes.science.psu.edu/	49	Contain also 118 entities similar to miRNAs
miRNEST	http://rhesus.amu.edu.pl/mirnest/copy/browse.php	398	An integrative miRNA resource

**Table 3:** High-confidence miRNA targets in *Hordeum vulgare* together with their experimentally verified or supposed biological functions in higher plants.

miRNA	mRNA Target(s) in *Hordeum vulgare*	Known Biological Function(s) of miRNA in Plant Species and Further Notes	References
miR156a	SBP-box gene family member	Inflorescence morphogenesis regulation in tomato (*Solanum lycopersicum*) plants; male fertility regulation in thale cress (*Arabidopsis thaliana*) plants	[114,115,116]
miR156b			
miR159a	MYB family transcription factor;lectin-like receptor kinase	Ensure normal growth via regulation of *GAMYB* genes	[117,118,119]
miR159b	MYB family transcription factor;		
miR166a	START domain-containing protein; MATE domain-containing protein; class III HD-Zip protein 8	Shoot apical meristem and vascular differentiation, leaf and root development; evolutionarily conserved stress biomarker in land plants—drought, salinity, temperature, biotic stress	[120,121]
miR166b			
miR166c			
miR168-5p	receptor-like protein kinase 5 precursor	Function in plants is unclear but targets many important mammalian transcripts (123 in total), including the gene for Low-density lipoprotein receptor adaptor protein 1 (*LDLRAP1*, also known as *ARH*))	[122]
miR171-3p	scarecrow transcription factor family protein	Regulation of germination and seedling growth in Tibetan hull-less barley (*Hordeum vulgare* L. var. *nudum*); drought tolerance by regulation of flavonoid biosynthesis genes in rice	[113,123]
miR397a	laccase precursor protein; transporter family protein;	Plant development; circadian regulation and plant flowering; cold response in thale cress (*Arabidopsis thaliana*)	[124,125]
miR399	rp1; ubiquitin-conjugating enzyme protein; pentatricopeptide	Salt stress response and flowering regulation in thale cress (*Arabidopsis thaliana*)	[126,127]
miR444a	FAD-binding domain of DNA photolyase domain-containing protein; DnaK family protein; alpha-taxilin; MADS-box family gene with MIKCc type-box; pentatricopeptide; WD domain, G-beta repeat domain-containing protein	Regulation of nitrate signaling pathway in nitrate-dependent root growth, nitrate accumulation, and phosphate-starvation responses in rice (*Oryza sativa*); antiviral pathway in rice; regulation of brassinosteroids synthesis in rice	[128,129,130]
miR444b	MADS-box family gene with MIKCc type-box; methyltransferase; zinc finger, C3HC4 type domain-containing protein		
miR1120	An enzyme of the cupin superfamily protein; retrotransposon protein; tesmin/TSO1-like CXC domain-containing protein; WD domain, G-beta repeat domain-containing protein; CCR4-NOT transcription factor; glycosyltransferase family 43 protein; amine oxidase-related; Divergent PAP2 family domain-containing protein	Early anther development in wheat (*Triticum aestivum*). miR1120 in barley has many diverse mRNA targets, however, it is questionable, if this miR1120 is a true miRNA (originating from hairpin RNA precursor), as the miR1120 gene region in barley displays almost 80% sequence similarity to the short transposon element DNA/TcMar-Stowaway	[93,131]
miR1436	pseudogene	Various stress responses in *Cestrum nocturnum* L. and *Cestrum diurnum* L.	[132]
miR5048a	cysteine-rich receptor-like protein kinase precursor	Wheat (*Triticum aestivum*) grains development regulation	[133]
miR5049c	modifier of rudimentary protein; auxin-induced protein 5NG4; Spc97/Spc98 family protein; protein kinase domain-containing protein; OsWAK receptor-like protein kinase	Hormone, stress (heat, drought, salinity, and excess boron), and light responsiveness in barley (*Hordeum vulgare* L.)	[67]
miR5049f	resistance protein; transcription factor-related; WD domain, G-beta repeat domain-containing protein; TBC domain-containing protein;	Regulation of salt adaptation in *Hordeum bulbosum*	[75]
miR6197	DUF26 kinase; exosome complex exonuclease rrp4	Boron stress response regulation in barley (*Hordeum vulgare*)	[83]
miR6201	C4-dicarboxylate transporter/malic acid transport protein	Cadmium stress response regulation in wheat (*Triticum aestivum*)	[134]
